# Healthcare providers' perception and knowledge of the use of artificial intelligence in healthcare service delivery in the Limbe and Buea Health Districts: a cross-sectional study

**DOI:** 10.3389/fdgth.2025.1575633

**Published:** 2025-10-01

**Authors:** Oliver Itoe, Francis Desire Tatsinkou Bomba, Odette Dzemo Kibu, Innocentia Ginyu Kwalar, Elvis Asangbeng Tanue, Denis Nkweteyim, Madeleine L. Nyamsi, Peter L. Achankeng, Christian Tchapga, Justine Ayuk, Moise Ondua, Patrick Jolly Ngono Ema, Maurice Marcel Sandeu, Gregory Eddie Halle-Ekane, Jude Dzevela Kong, Dickson Shey Nsagha

**Affiliations:** ^1^ Department of Public Health and Hygiene, Faculty of Health Sciences, University of Buea, Buea, Cameroon; ^2^ Department of Biomedical Sciences, Faculty of Health Sciences, University of Buea, Buea, Cameroon; ^3^ DigiCare Cameroon Consortium, University of Buea, South West Region, Cameroon; ^4^ Department of Computer Sciences, Faculty of Sciences, University of Buea, Buea, Cameroon; ^5^ Department of Women and Gender Studies, Faculty of Social and Management Sciences, University of Buea, Buea, Cameroon; ^6^ Department of Microbiology and Infectious Diseases, School of Veterinary Medicine and Sciences, University of Ngaoundéré, Ngaoundéré, Cameroon; ^7^ Department of Genetics and Biostatistics, School of Veterinary Medicine and Sciences, University of Ngaoundéré, Ngaoundéré, Cameroon; ^8^ Department of Obstetrics and Gynaecology, Faculty of Health Sciences, University of Buea, Buea, Cameroon; ^9^ Department of Mathematics & Statistics, York University, Toronto, ON, Canada

**Keywords:** artificial intelligence, healthcare, healthcare provider, perception, Cameroon

## Abstract

**Background:**

Artificial Intelligence (AI) in healthcare is rapidly growing in recent years, and has substantially improved the quality of care and health outcomes of patients. Understanding healthcare providers' perception and knowledge of AI in healthcare is crucial for its effective adoption, and its use. This study aimed to determine healthcare providers' awareness, assess their knowledge of healthcare AI, assess their perceived benefits, readiness to adopt AI in healthcare in Limbe and Buea Health Districts.

**Methods:**

A hospital-based cross-sectional study was conducted using a multi-staged sampling technique that recruited participants from seven hospitals in Limbe and Buea Health Districts. A questionnaire designed on koboCollect was used for data collection through face-to-face interviews from 494 participants recruited through a multi-stage sampling technique. The data was analyzed using SPSS version 26 where descriptive statistics and logistic regressions were done to determine the factors associated with readiness to adopt AI in healthcare. A *P*-value of <0.05 at 95% CI was considered statistically significant.

**Results:**

A total of 494 participants were recruited into the study with a mean age of 32.6 ± 7.5 years, the majority 355 (71.9%) were females, 448 (90.7%) had attained tertiary education and the highest proportion 295 (59.7%) were Nurses. The study revealed that 373 (75.5%) were aware of the use of AI in healthcare, 261 (52.8%) had used AI tools, 213 (43.1%) had good knowledge of healthcare AI, 283 (57.3%) had good perception of its benefits and 230 (46.6%) were ready to adopt its use. Those who had access to AI tools were about 5 times more ready to adopt AI use (AOR: 4.5, CI: 3.05–6.72, *p*: <0.001). The main challenges reported were job displacement, lack of understanding of AI, and limited access to quality health data. A majority of 465 (94.1%) believed training is important to effectively use AI in healthcare.

**Conclusion:**

Healthcare providers' awareness and perceived benefits of AI use in healthcare were good, the knowledge was below average, and an average of the population were ready to adopt AI. Despite the benefits of AI, most of them fear AI will replace their jobs and believe training is important for the effective adoption of AI in healthcare.

## 1 Introduction

Artificial intelligence (AI) started in early 1950s when Alan Turing developed the Turing test whereby he asked that, “can machines think?” (1) and considered that machines can learn like a child. AI has evolved from the fundamental AI algorithms to machine learning and deep learning algorithms over the years (2). Many definitions of AI exist, but Aiken and Epstein defined AI as a branch of science and technology that allows intelligent computers and computer programs to perform tasks that would typically need human intelligence (3). It is therefore, the imitation of human intellect in computers to carry out tasks like learning, reasoning, problem solving, speech recognition, and decision-making that normally need human intelligence due to its ability to access and analyse large input data (4, 5).

Its application in healthcare, known as healthcare AI has experienced rapid growth in the past decades due to the promising benefits it has offered to the healthcare industry that generates vast amount of health data (6) which can be used to train these machines. This rapid growth has led to patients accessing healthcare from the comfort of their homes (7). The emergence of the coronavirus disease greatly transformed the fundamental building blocks of global health system forcing healthcare stakeholders to adopt digital technologies in healthcare (8). AI has revolutionized the health sector and has substantially improved the quality of services rendered to patients from disease prevention, early detection of diseases, diagnosis, treatment, adherence to treatment, and follow-up of care (9–11). AI can be more cost-effective, accurate, and precise when compared with humans due to its ability to analyse large input data and make evidence-based decision (12–14) as such can reduce patients waiting time, medical errors and length of stay in hospitals. AI has played a crucial role in healthcare through big data analysis, diagnosis, precision medicine, electronic health records, virtual patient care, administrative duties, and research and drug development (15) as well as patient monitoring using wearable sensor devices that have allowed for prompt treatment when need arises (16).

Khosla in 2012, reported that AI will be incorporated into about 80% of hospitals by 2025 and will perform about 90% of tasks that physicians were currently doing (17). Despite the benefits of AI, its application in healthcare especially in Low and Middle Income Countries is still very lacking (18) due to a number of factors such as the absence of regulatory guidelines, unavailability of easy to use AI tools, inappropriate infrastructure and poor internet connections. There exist a lot of obstacles to the use of AI in healthcare. including standardization gaps, unclear legal liability, and ethical concerns (19) as well as misconception about how it will affect the health sector, the patient and the government especially in low- and middle-income countries where data generated are usually disorganized, fragmented, and dispersed making it difficult to be used in real time situations (20, 21). The health sector which is very heavily regulated (22) needs strict rules and guidelines to validate the use of AI tools in healthcare. This requires that the personnel have the knowledge and skills to use and interpret results generated by AI tools (23) which is a major shortcoming in the context of LMICs. The fear that AI will replace jobs (24) has resulted to resistance to the adoption of AI. The difficulty and complexity in understanding how AI tools operate (25) have compromised its trust and validity. AI can breech patient-provider relationship, lacks empathy, emotion and cannot be appropriate in complex health conditions where the inputs from many disciplines are required to come out with a solution (26). It is therefore important to understand that to effectively adopt AI in healthcare, its benefits must be tangible followed by appropriate ethical and legal regulatory standards (27) and healthcare leaders have a role to play when it concerns setting up the standards of health practices in the health system.

The readiness to adopt AI in healthcare can be influenced by the willingness and optimism of healthcare providers about its use as well as the technological, organisational, and environmental (TOE) factors. The TOE framework (28) that was established by Tornatsky and Fleischer in 1990 was considered appropriate and adapted for the study and was used to assess healthcare providers' readiness to adopt AI in healthcare.

The aim of this study was to determine the proportion of awareness and knowledge of the use of AI, perceived benefits, readiness to adopt AI, and perceived challenges in the implementation of AI in healthcare service delivery among healthcare workers in the Limbe and Buea Health Districts of Cameroon.

## 2 Materials and methods

### 2.1 Study design and population

A hospital based cross-sectional study, targeting healthcare providers, was conducted in the Limbe and Buea Health Districts of Cameroon from February 2024 to June 2024.

### 2.2 Selection criteria

The inclusion criteria were healthcare providers aged 18 years and above, and who have at least six months of working experience while healthcare providers who were sick and mentally unstable were excluded from the study. The different categories of healthcare providers who participated in the study were medical doctors, nurses, laboratory scientists, radiologists, pharmacists, midwives and nutritionists.

### 2.3 Sample size determination and sampling

From the personnel registry of the Limbe Health District, the estimated number of personnel (1,139) was used to calculate the sample size of 296 while for Buea, 391 health personnel gave 198 using the Yamane's (1967) formula (29). The two were summed up to 494 participants as minimum for the study. A multi-staged sampling technique was used to randomly selected the 296 participants from five health facilities in Limbe Health District (Regional Hospital Limbe, District Hospital Limbe, Sub-Divisional Hospital Limbe, Bota Polyclinic, and PCC Health Centre Limbe) and the 198 participants from two health facilities in Buea Health District (Regional Hospital Buea, and Mount Mary Hospital Buea) to participate in the study.

### 2.4 Ethical consideration

Ethical clearance was obtained from the Institutional Review Board (IRB) of the Faculty of Health Sciences (FHS) No 2024/2331-01 of the University of Buea followed by administrative authorizations to carry out the research from the seven selected health facilities.

### 2.4 Data collection

A questionnaire, adapted from the Shinners artificial intelligence perception tool (30) and from the existing reviewed literature was designed and deployed on KoboCollect v2023.2.4 for data collection through face-to-face based interview with the use of smart phones. Pretesting of fifteen questionnaires was done in the Sub-Divisional Hospital Bonadikombo and PCC eye clinic Limbe to assess the feasibility and correctness of the data collection tool in responses to obtaining relevant data for the attainment of the specific objectives of the study. The questionnaire advised potential respondents that consent was implied by completing the survey and submitting the questionnaire into the online KoboCollect application while anonymity and confidentiality of the respondents were respected throughout the interview.

The scoring was done based on a set of questions each pertaining to knowledge, perception, and readiness. The correct/appropriate response was scored as “1” and incorrect/inappropriate response score as “0” which were summed up and a cut-off for each was determined. Scores greater than the cut-off score were considered good/ready and scores less than or equal to the cut-off score were considered poor/not ready.

### 2.5 Data management and analysis

Completely filled questionnaires submitted online into Kobo Collect were later downloaded onto MS Excel, then cleansed and stored in a safe external storage device. The data were then exported to IBM SPSS statistics v26 for analysis. Descriptive statistics was done for discrete variables and presented as means and standard deviations while categorical variables were presented as frequency tables and charts and logistic regression was used to analyse the association of socio-demographic characteristics of the participants with their readiness to adopt AI in healthcare service delivery. Variables with that from the binary analysis with a *P*-value of <0.02 at 95% confidence interval were imported into the logistic regression model. A *P*-value of <0.05 at 95% confidence interval was considered statistically significant.

## 3 Results

### 3.1 Socio-demographic characteristics of the participants

A total of 494 healthcare providers were recruited in the study whose mean age was 32.6 ± 7.5 years and mean duration of working experience was 6.0 ± 5.1 years. The highest proportion of the participants of the study as shown in Table 1 was recorded from Regional Hospital Limbe being 200 (40.5%) followed by Regional Hospital Buea 109 (22.1%) and the lowest was from Presbyterian Health Center Down Beach Limbe. The majority of the respondents were made up of females 355 (71.9%), Nurses 295 (59.7%), those who had attained a tertiary level of education 488 (90.7%), and owned smart phones 485 (98.2%). However, only a few 266 (53.8%) of them have had access to an AI tool.

**Table 1:** Socio-demographic characteristics of the participants.

Variables	Categories	Number (%)
Health facilities	Bota Poly Clinic	18 (3.6)
	CMA Limbe	32 (6.5)
	District Hospital Limbe	39 (7.9)
	Mount Mary Hospital Buea	80 (16.2)
	Presbyterian Health Center Down Beach	16 (3.2)
	Regional Hospital Buea	109 (22.1)
	Regional Hospital Limbe	200 (40.5)
	Total	494 (100)
Sex	Female	355 (71.9)
	Male	139 (28.1)
	Total	494 (100)
Profession	Laboratory Scientist	82 (16.6)
	Medical Doctor	41 (8.3)
	Midwife	40 (8.1)
	Nurse	295 (59.7)
	Nutritionist	2 (0.4)
	Pharmacist	27 (5.5)
	Radiologist	7 (1.4)
	Total	494 (100)
Level of education	Secondary	46 (9.3)
	Tertiary	448 (90.7)
	Total	494 (100)
Own any Digital tools	No	9 (1.8)
	Yes	485 (98.2)
	Total	494 (100)
Access to AI tools like ChatGPT	No	228 (46.2)
	Yes	266 (53.8)
	Total	494 (100)

### 3.2 Awareness of artificial intelligence use in healthcare among healthcare providers

**Figure 1:**
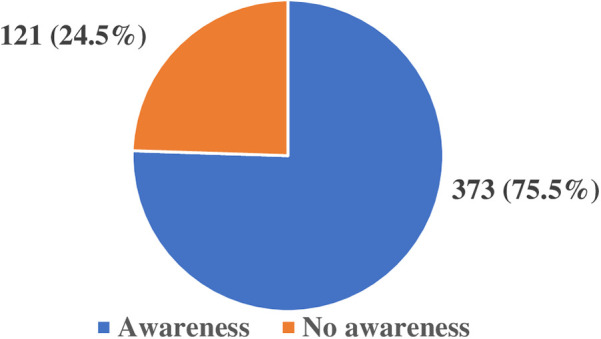
Healthcare providers' awareness of the use of artificial intelligence in healthcare.

From the results of the study a total of 409 (82.8%) participants reported having heard of AI before and out of this number, 373 (75.5%) were aware that AI can be used in healthcare service delivery and only 261 (52.8%) of them had used one or more AI tools in their practices. Figure 1 shows the proportion of healthcare providers' awareness of AI use in healthcare.

### 3.3 Assessment of healthcare provider's knowledge of healthcare artificial intelligence

**Figure 2:**
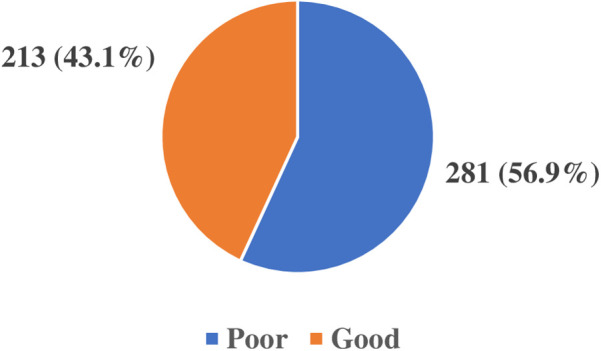
Healthcare providers' knowledge of the use of artificial intelligence in healthcare.

The healthcare providers' knowledge of the use of AI in healthcare was assessed from a set of four questions which had a total score of 11points, with a cut-off of 6/11 points and above considered as having good knowledge while from 5/11 and below poor knowledge. The results as shown in Figure 2 revealed 213 (43.1%) had good knowledge on AI use in healthcare and 281 (56.9%) had poor knowledge.

### 3.4 Healthcare providers' perception of the benefits of artificial intelligence in healthcare

**Figure 3:**
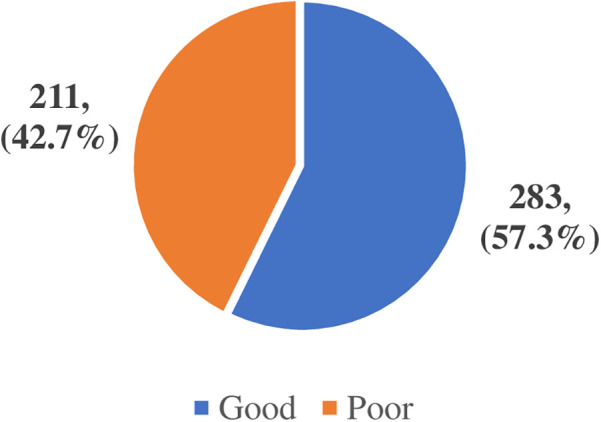
Healthcare providers' perception of the benefits of artificial intelligence in healthcare.

The perception of the benefits of AI in healthcare of the participants was categorized as good or poor following a scoring of 10 pts from a set of three questions. A cut-off of 6 was categorized as good perception of the benefits of AI in healthcare while from 5 and below was categorized as poor and summarized in Figure 3.

### 3.5 Healthcare providers' readiness to adopt artificial intelligence in healthcare

**Figure 4:**
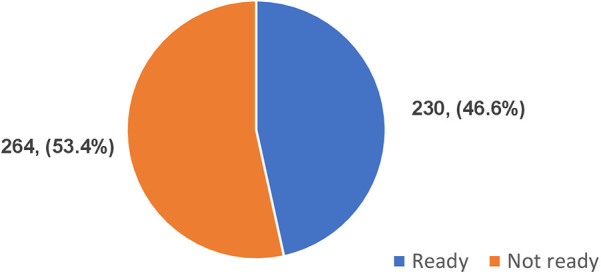
Healthcare providers' readiness to adopt artificial intelligence in healthcare.

With respect to adopting AI in healthcare, a set of three questions were scored, based on their willingness, optimism and the TOE framework and on a scale of 13 with a cut-off score of 7/13. Scores greater than 7 were categorized as ready and from 7 and below were considered not ready. The study revealed 230 (46.6%) readiness to adopt AI in healthcare while 264 (53.4%) not ready as shown in Figure 4.

### 3.6 Factors associated with the readiness to adopt AI

Tables 2, 3 shows the factors that were significantly associated with the readiness to adopt AI in healthcare at multivariate analysis using the multiple logistic regression model. The odds of those with tertiary education being ready to adopt AI in healthcare service delivery were 0.1 (95% CI: 1.027–4.888, *P* = 0.043) times lower than the odds of those of secondary education. The odds of those who had access to AI tools were 4.5 times (95% CI: 3.053–6.715, *P* ≤ 0.001) ready to adopt AI tools in healthcare.

**Table 2:** Independent analysis of factors associated with readiness to adopt AI.

Variables	Categories	Total number	Readiness to adopt AI		COR	95% CI for COR		*P*-value
			Yes (%)	No (%)		Lower	Upper	
Sex	Female	355	153 (31.0)	202 (40.9)	1.6	1.105	2.434	0.014
	Male	139	77 (15.6)	62 (12.6)	1			
	Total	494	230 (46.6)	264 (53.4)				
Age group (years)	20–29	194	86 (17.4)	108 (21.9)	1.3	0.464	3.796	0.598
	30–39	212	110 (22.3)	102 (20.6)	1.8	0.631	5.123	0.273
	40–49	72	28 (5.7)	44 (8.9)	1.1	0.341	3.243	0.918
	50+	16	6 (1.2)	10 (2.0)	1			
	Total	494	230 (46.6)	264 (53.4)				
Profession	Radiology staff	7	5 (1.0)	2 (0.4)	1.4	0.248	8.372	0.683
	Pharmacy staff	27	17 (3.4)	10 (2.0)	1	0.358	2.685	0.97
	Nutritionist	2	1 (0.2)	1 (0.2)	0.6	0.034	9.911	0.705
	Midwife	40	24 (4.9)	16 (3.2)	0.9	0.353	2.121	0.752
	Nurse	295	112 (22.7)	183 (37.0)	0.4	0.179	0.695	0.003
	Laboratory staff	82	45 (9.1)	37 (7.5)	0.7	0.325	1.516	0.367
	Medical Doctor	41	26 (5.3)	15 (3.0)	1			
	Total	494	230 (46.6)	264 (53.4)				
Level of education	Secondary	46	10 (2.0)	36 (7.3)	0.3	0.139	0.594	0.001
	Tertiary	448	220 (44.5)	228 (46.2)	1			
	Total	494	230 (46.6)	264 (53.4)				
Years of experience	1–10years	418	193 (39.1)	225 (45.5)	6.9	0.851	55.355	0.071
	11–20years	67	36 (7.3)	31 (6.3)	9.3	1.101	78.459	0.041
	21+	9	1 (0.2)	8 (1.6)	1			
	Total	494	230 (46.6)	264 (53.4)				
Know any digital tool	Yes	485	230 (46.6)	255 (51.6)	0.4	0.001		0.999
	No		0 (0.0)	9 (1.8) %	1			
	Total	494	230 (46.6)	264 (53.4)				
Have access to AI tools	Yes	266	169 (34.2)	97 (19.6)	4.8	3.245	7.011	<0.001
	No	228	61 (12.3)	167 (33.8)	1			
	Total	494	230 (46.6)	264 (53.4)				

**Table 3:** Multivariate analysis of factors associated with readiness to adopt artificial intelligence in healthcare.

Variable	Categories	Number	Readiness to adopt AI			95% CI for AOR		*P*-value
			Yes number (%)	No number (%)	AOR	Lower	Upper	
Level of education	Tertiary	448	220 (44.5)	228 (46.2)	0.043	1.027	4.886	0.043
	Secondary	46	10 (2.0)	36 (7.3)	1			
	Total	494	230 (46.6)	264 (53.4)				
Work experience (year)	11–20years	67	36 (7.3)	31 (6.3)	0.168	0.019	1.484	0.109
	1–10years	418	193 (39.1)	225 (45.5)	1.674	0.939	2.986	0.081
	21+	9	1 (0.2)	8 (1.6)	1			
	Total	494	230 (46.6)	264 (53.4)				
Have access to AI tools	Yes	266	169 (34.2)	97 (19.6)	4.528	3.053	6.715	<0.001
	No	228	61 (12.3)	167 (33.8)	1			
	Total	494	230 (46.6)	264 (53.4)				

### 3.7 Healthcare providers' perceived challenges with the use of artificial intelligence in healthcare

The results in Table 4 showed that a good proportion of the participants 296 (59.9%) had never had any formal or informal form of training in AI while 178 (36.0%) have had informal training. The majority 349 (70.6%) were very concerned about the ethical implications of the use of AI in health as issues of confidentiality, patient's privacy and lack of empathy in AI tools while the primary challenges were fear of job displacement 308 (27.5%), lack of knowledge of the functionality of AI tools 277 (24.8%), and limited access to quality health data 217 (19.4%).

**Table 4:** Challenges perceived by healthcare providers with the use of AI in healthcare.

Variable	Categories	Number (%)
Received any form of training on the use of AI	Yes	178 (36.0)
	No	296 (59.9)
	Not sure	20 (4.0)
	Total	494 (100)
Importance of AI training to healthcare professionals	Extremely important	114 (23.1)
	Moderately important	83 (16.8)
	Quite important	216 (43.7)
	Slightly important	52 (10.5)
	Not important	29 (5.9)
	Total	494 (100)
Concerns about ethical implications of using AI	Yes	349 (70.6)
	No	91 (18.4)
	Not sure	54 (10.9)
	Total	494 (100)
Primary challenges of AI in healthcare	Limited access to quality health data	217 (19.4)
	Job displacement of personnel	308 (27.5)
	Ethical consideration	211 (18.9)
	Lack of understanding of AI technology	277 (24.8)
	Legal and regulatory issues	88 (7.9)
	Others	17 (1.5)
	Total	1,118 (100)^a^

^a^ Multiple response question.

## 4 Discussions

The use of artificial intelligence in healthcare which is in its initial phase in this region is very much exiting and promising owing to the many benefits it has in the health sector. The findings of this study have provided insights into healthcare providers' perception and knowledge of the use of artificial intelligence in healthcare service delivery which is relevant for developing future strategies in Public health to facilitate the adoption and use of AI in healthcare.

Most of the participants had heard about artificial intelligence before and fewer of them had actually used either AI tools from the application on their smart phones, or wearable sensor devices. A study by Samyuktha et al. (31) carried out in India in 2020 on awareness and knowledge about artificial intelligence in healthcare among doctors showed an awareness of about 55% of artificial intelligence in healthcare. The results also revealed that only 43.1% of them had good knowledge and understanding of AI use in healthcare though many did not understand the full applicability of AI in their practices. This higher awareness and knowledge can be attributed to the rapidly growing technology, and information and communication tools like smart phones with the stable internet connection, allowing more healthcare workers to be informed about AI at anytime and anywhere. This is supported by the fact that 98.2% of the participants owned at least a smart phone giving them access to vast information around the world. The highest proportion of the respondents were of the age group 30–39 years accounting for the younger generation being more aware and engaged with technologies and their uses than the older generation. Due to the fact that a vast majority of the respondents had attained tertiary education which when combined with the use of smart phones they can easily get access to information and gain more knowledge on AI use in healthcare. A study carried out by Catalina et al. (32) on knowledge and perception of primary care healthcare professionals on the use of artificial intelligence as a healthcare tool in 2023, revealed an awareness of 85.7% which is higher compared with that of this study. This can be due to the fact that the use of artificial intelligence is more integrated into their healthcare system than in the Health Districts of Limbe and Buea. The healthcare providers' perception of the benefits of AI in healthcare from the study showed that a good proportion of the respondents had good perception about AI's benefits in healthcare as it can enhance patient management, assist in evidence-based decision making, diagnostic precision and accuracy, reduction in patient waiting time and medical errors and as such reduction in health cost both to the patient and the personnel and reduced length of stay in the hospital.

With respect to the readiness to adopt AI, few of the respondents were ready to adopt AI in their practices. A study by Ogolodom et al. (33) in Nigeria, reported that most of the respondents agreed that AI can be incorporated into medical specialties and were ready to use it. Nonetheless, its adoption in healthcare has to be strictly regulated and validated to ensure compliance and to avoid unnecessary bias.

This study's results revealed that the perceived fear that AI will replace healthcare providers in their jobs, lack of knowledge of how AI functions and limited access to quality health data were the major challenges in the implementation of AI in healthcare among others. A similar report done in Saudi Arabia by Serbaya et al. (34) revealed that 58% of healthcare workers expressed fear about job displacement. Ogolodom et al. (33) in Nigeria, agreed that healthcare workers are at risks of being replaced in their jobs which is consistent with this study. Quite a good number of the respondents are very concerned about the ethical issues of using machines to perform tasks of care to patients as they think the relationship between the patient and the health care providers may be breeched as machines can never provide emotional support, empathy and have no feelings as such AI may dehumanize healthcare service delivery (35) and only a few of the respondents trusted AI driven decision-making. WHO has developed six ethical principles to guide the development of AI tools. Ethical principles guiding AI in healthcare research are deeply rooted in the foundational principles of medical research ethics.

Training was an important facilitator mentioned by the health care provider to adopt AI in health care service. According to Catalina et al. (32), 65.8% of healthcare professionals also indicated that they had not received any training and 91.4% would like to receive training on AI which is similar with the results obtained in this study.

## 5 Limitations of the study

Limited scope of the study: The study was focused on two Health Districts of Cameroon and mainly in the urban areas which may limit the generalizability of the findings to other Regions or beyond Cameroon. Studies from rural or primary healthcare settings are required for a more representative view. Furthermore, the study relies on self-reported awareness and use of AI, which may be influenced by social desirability and participant might have overreported on their digital skills.

## 6 Conclusions

This study found out that healthcare providers were aware that artificial intelligence can be used in healthcare, though their knowledge of the functionality of AI in healthcare is slightly below average. They were very optimistic and willing to adopt AI in their practices. This result will guide the implementation of AI taking into consideration the challenges and concern of the health care providers. Training and sensitisation is necessary to facilitate the use of AI in health care.

## Data Availability

The raw data supporting the conclusions of this article will be made available by the authors, without undue reservation.
